# A novel miRNA-4484 is up-regulated on microarray and associated with increased MMP-21 expression in serum of systemic sclerosis patients

**DOI:** 10.1038/s41598-019-50695-y

**Published:** 2019-10-03

**Authors:** Marta Rusek, Małgorzata Michalska-Jakubus, Małgorzata Kowal, Jerzy Bełtowski, Dorota Krasowska

**Affiliations:** 1 Department of Dermatology, Venereology and Pediatric Dermatology, Laboratory for Immunology of Skin Diseases, Medical University of Lublin, Lublin, Poland; 2 Department of Pathophysiology, Medical University of Lublin, Lublin, Poland

**Keywords:** Transcriptomics, Diagnostic markers

## Abstract

Systemic sclerosis (SSc) is a complex, heterogeneous connective tissue disease, characterized by fibrosis and ECM deposition in skin and internal organs, autoimmunity, and changes in the microvasculature. Profiling of circulating miRNAs in serum has been found to be changed in pathological states, creating new possibilities for molecular diagnostics as blood-based biomarkers. This study was designed to identify miRNAs that are differentially expressed in SSc and might be potentially contributing to the disease etiopathogenesis or be used for diagnostic purposes. Thus, we compared the expression pattern of multiple miRNAs in serum of 10 SSc patients to 6 healthy controls using microarray analysis, and RT-qPCR to confirm the obtained results. In addition, bioinformatics analysis was performed to explore miRNAs target genes and the signaling pathways that may be potentially involved in SSc pathogenesis. Our study shows a different expression of 15 miRNAs in SSc patients. We identified that miR-4484, located on chromosome 10q26.2, was an 18-fold up-regulated in SSc patients compared to a control group. Bioinformatics analysis of the miR-4484 target genes and the signaling pathways showed that it might be potentially involved in the TGF-β signaling pathway, ECM-receptor interaction, and metalloproteinases expression. Based on the chromosomal location, the most interesting target gene of miR-4484 may be MMP-21. We found that the expression of MMP-21 significantly increased in SSc patients compared to healthy subjects (*P* < 0.05). Our results suggest that miR-4484, and MMP-21 might be novel serum biomarkers that may correspond to pathological fibrosis in SSc, but it needs to be validated in further studies.

## Introduction

Systemic sclerosis (SSc) is a complex, heterogeneous connective tissue disease (CTD), characterized by immune abnormalities, microvasculopathy with capillary loss and excessive extracellular deposition with subsequent fibrosis of the skin and internal organs^1^. This results in life-threatening complications, such as interstitial lung disease, pulmonary hypertension, or heart failure^2^. Currently, the exact etiology of the disease remains unknown; however, infection, immune activation by cancer, or dysbiosis might be potential triggers on the genetic background^1,3^. Moreover, this disorder is observed mainly in female patients.

The pathological steps in the disease process are also unclear. They seem to include an autoimmune attack, vascular damage, and tissue inflammation that finally trigger abnormal activation of fibroblasts with enhanced collagen deposition into the extracellular matrix (ECM)^4^. The best-studied pathways, which play a role in driving collagen production, and promoting fibrotic matrix deposition are the transforming growth factor-β (TGF-β), canonical Wnt/β-catenin, and Toll-like receptor (TLR) signaling^3,5^. Moreover, matrix metalloproteinases (MMPs) are essentially involved in connective tissue remodeling, since they are capable of degrading ECM and basement membrane proteins, as well as modulate inflammatory reaction by cleaving cytokines, their receptors and growth factors^6^. Evidence suggests that fibrotic responses may also be modulated by transcriptional activators, suppressors or cofactors^4^.

MicroRNAs (miRNAs) are endogenous, short (19–25 nucleotides long), non-coding single-stranded RNAs molecules that are post-transcriptional regulators of many biological processes involved in almost all aspects of cell physiology^7^. Since there are about 1000 miRNAs in the human genome, which may regulate approximately one-third of the human protein-coding genes^5^, miRNAs are the most abundant class of regulators^8^. They act by binding to complementary sequences of their target mRNAs to control protein expression in physiological and pathophysiological conditions^9^. One miRNA can target several mRNAs, and many miRNAs can also target one mRNA^4^. Generally, miRNA–mRNA interaction takes place in the 3′-untranslated region (3′-UTR) of mRNA that negatively regulates gene expression by promoting protein production^10^. However, sometimes the miRNA–mRNA interaction lies in the 5′-UTR, leading to enhancement of translation^4^. The profiling of miRNAs has made a significant impact on biomedical research, particularly the gene expression changes in pathological states. Recently, miRNAs have attracted attention as blood-based biomarkers and potential pathogenic contributors in the pathogenesis of various autoimmune diseases, including SSc^5,9^. Many reports suggest that miRNAs and their targets are aberrantly expressed in skin and fibroblasts of SSc patients compared to healthy tissues, thus making them potential novel players in SSc fibrosis^4^. They are thought to regulate numerous fibrotic-related genes; however, their exact role in SSc pathogenesis remains unclear, and studies with a broad profiling of circulating miRNAs are scarce^11^. Several miRNAs have also been shown to be increased in sera of SSc patients, which indicates them as potential diagnostic biomarkers^7,11^.

The aim of this study was to examine a comprehensive profiling of miRNAs in serum of SSc patients and healthy controls to identify the differentially expressed miRNAs by using miRNA microarray analysis and validation of selected miRNAs by RT-qPCR. In addition, we performed bioinformatics analysis to explore target genes for differentially expressed miRNAs, their possible signaling pathways potentially involved in SSc pathogenesis, and a gene ontology term enrichment analysis to determine signaling pathways and biological processes associated with target genes.

## Results

### Expression profiling of miRNAs in SSc patients and healthy control serum samples

**Figure 1:**
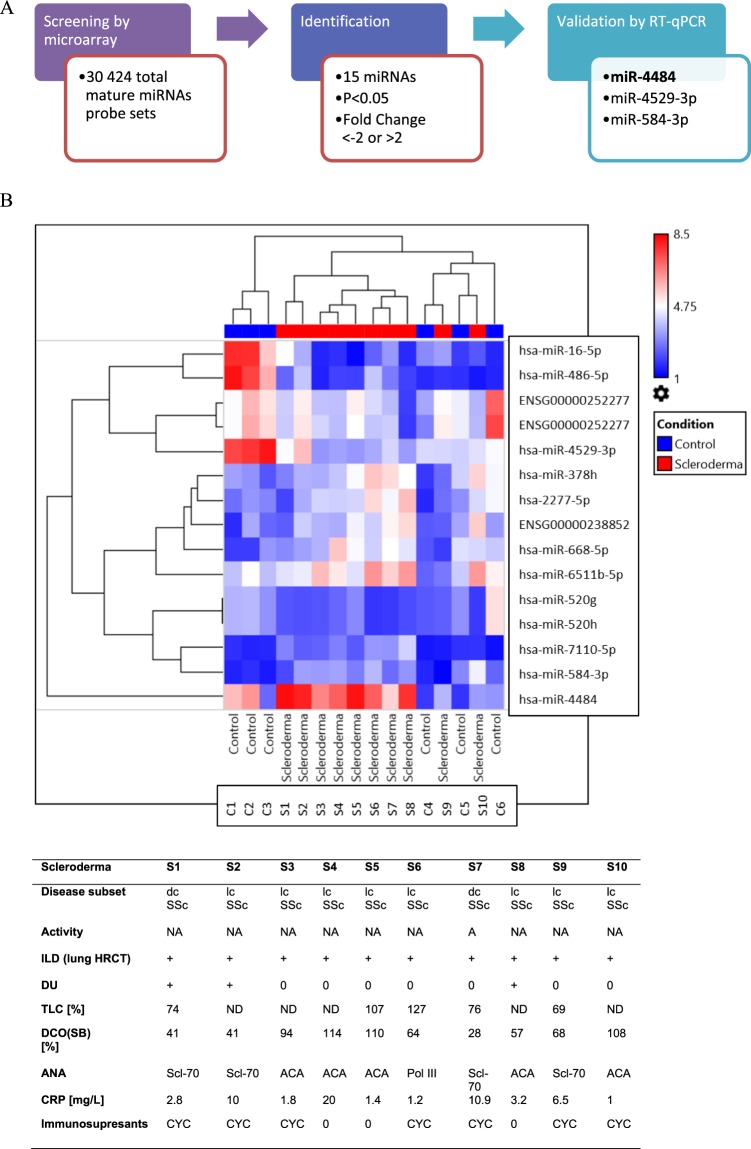
Screening and identification of the miRNAs that are differentially expressed in SSc patients. (**A**) The pattern of the study design; (**B**) A heat map based on the clustering of miRNAs in SSc patients and healthy controls along with selected clinical and laboratory features of each SSc patient. MiRNAs are indicated vertically on the right. Each column represents a sample group; each row represents a miRNA. The color scale indicates the relative expression of miRNAs, where red shows higher expression and blue lower expression. lcSSc, limited cutaneous SSc; dcSSc, diffuse cutaneous SSc; A, active; NA, not active; + , present; 0, absent; ND, not done; DCO(SB), diffusing capacity for carbon monoxide, single-breath-measurements; TLC, total lung capacity; ANA, anti-nuclear antibodies; ILD, interstitial lung disease; DU, digital ulcers; Scl-70, anti-topoisomerase antibodies; ACA, anti-centromeric antibodies; Pol III, anti-polymerase III antibodies, CRP, C-reactive protein, CYC, cyclophosphamide.

We performed a miRNA microarray on serum samples from 10 SSc patients and 6 healthy controls to identify dysregulated miRNAs (Fig. 1A). By using the screening criteria of significant P value (<0.05) and fold change FC <−2 or >2, we have identified 15 differentially expressed miRNAs in serum of SSc patients *versus* healthy controls. Among them, 8 were up-regulated, and 7 were down-regulated (Table 1).

**Table 1:** Differentially expressed miRNAs by microarray in serum of SSc *vs* control group.

Transcript ID (Array Design)	Accession	ID	Scleroderma Avg (log2)	Control Avg (log2)	Fold Change	P-value
hsa-mir-520g	MI0003166	20535185	2,19	3,33	−2,21	0,0004
hsa-mir-520h	MI0003175	20535194	2,19	3,33	−2,21	0,0004
hsa-miR-7110-5p	MIMAT0028117	20526178	2,65	1,52	2,18	0,0008
hsa-miR-4484	MIMAT0019018	20518878	7,13	2,99	17,64	0,0023
hsa-miR-4529-3p	MIMAT0019068	20518930	3,79	6,05	−4,81	0,0025
ENSG00000238852	ENSG00000238852	20533559	4,06	2,83	2,34	0,0115
hsa-miR-584-3p	MIMAT0022708	20504313	3,18	1,54	3,12	0,0130
ENSG00000252277	ENSG00000252277	20533922	3,96	4,97	−2,01	0,0270
hsa-miR-16-5p	MIMAT0000069	20500128	2,43	4,55	−4,33	0,0280
hsa-miR-6511b-5p	MIMAT0025847	20525386	5,21	4,03	2,26	0,0299
hsa-miR-486-5p	MIMAT0002177	20503105	2,03	4,20	−4,50	0,0343
hsa-miR-668-5p	MIMAT0026636	20504554	4,06	2,84	2,32	0,0419
ENSG00000252277	ENSG00000252277	20533921	4,00	5,05	−2,07	0,0473
hsa-miR-378h	MIMAT0018984	20518842	4,25	3,21	2,06	0,0479
hsa-miR-2277-5p	MIMAT0017352	20512261	4,14	2,97	2,25	0,0484

15 microRNAs were changed <−2 or >2-fold; 8 were up-regulated and 7 were down-regulated.

After scanning the arrays, we performed a technical quality assessment of the data based on the signal intensity distribution results, background intensity levels, flagging of spots, and spike-in controls. In the next step, the microarray data was normalized, along with heat-maps with clustering assessment. The hierarchical clustering analysis showed that the miRNAs serum profile was almost perfectly separated for SSc patients and the control group (Fig. 1B). Based on the gene expression profile, the group of SSc patients and the control group are clustered on the bottom of the diagram. On the right, miRNA names are listed. The colors from blue to red represent the expression levels from lower to higher, respectively. Moreover, selected clinical and laboratory features of each SSc patient, along with the results of miRNA profiling are present in Fig. 1B.

### Validation of differentially expressed miRNAs in SSc patients and healthy control serum samples

**Figure 2:**
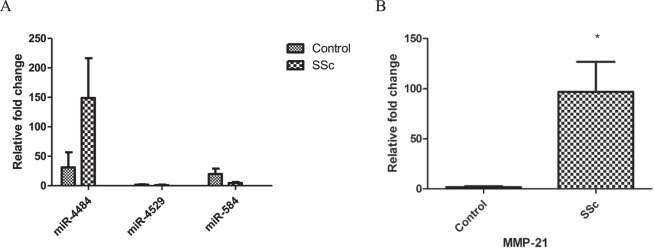
Validation of (**A**) aberrantly expressed miRNAs and (**B**) expression of MMP-21 in serum using RT-qPCR. The data are expressed as mean ± SD. All experiments were repeated at least 3 times. Data are presented as 2−∆ΔCT relative to the levels of RNU6 and ACTN, respectively. *Indicates P < 0.05.

The differential expression of three miRNAs (hsa-miR-4484, hsa-miR-4529-3p, hsa-miR-584-3p) selected based on the highest FC was confirmed by RT-qPCR. The results of the RT-qPCR were parallel to those of the microarray analysis, but we have not found the statistical significance (Fig. 2A).

### Target analysis of hsa-miR-4484

#### Bioinformatics analysis

Since hsa-miR-4484 demonstrated the highest (18-fold) change in up-regulation in SSc patients, compared to healthy subjects, we focused on hsa-miR-4484 in further analysis for potential targets. Using bioinformatics platforms, we found the potential target genes of hsa-miR-4484, which may be related to the pathology of SSc. TargetScan 7.0 has predicted 2617 transcripts with sites, containing a total of 3066 sites that might be regulated by miR-4484. Using a miRDB, we found that miR-4484 was predicted to regulate 33 targets, 60 targets in miRSearch, and 61 targets in miRTarBase. Moreover, using DIANA-microT v3.0, miR-4484 was predicted to regulate 1022 targets. We summarized the most interesting targets, which may play a role in SSc pathogenesis in Supplementary Table 1. Additionally, the full list of predicted targets for hsa-miR-4484 from Target Scan, miRDB, and miRTarBase is presented in Supplementary Table 2.

**Figure 3:**
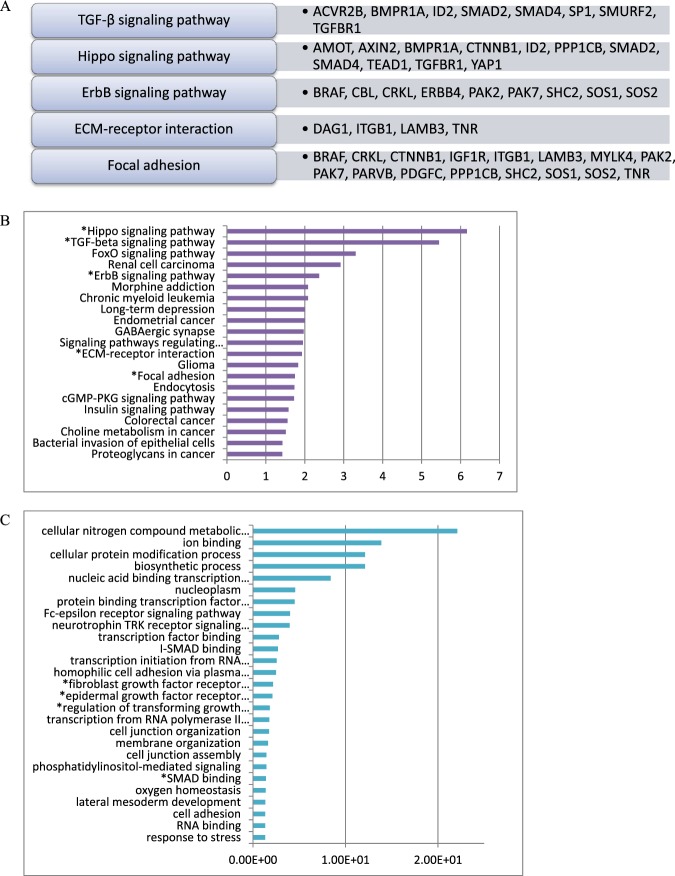
Enrichment analysis. (**A**) Predicted target genes for hsa-miR-4484; (**B**) The KEGG pathway analysis of hsa-miR-4484 gene targets in the serum of SSc patients compared to healthy controls; (**C**) The Gene Ontology related to the hsa-miR-4484 in the serum of SSc patients compared to healthy controls. The enrichment score is expressed as −log (P-value). *Indicates the possible role in pathogenesis in SSc.

**Figure 4:**
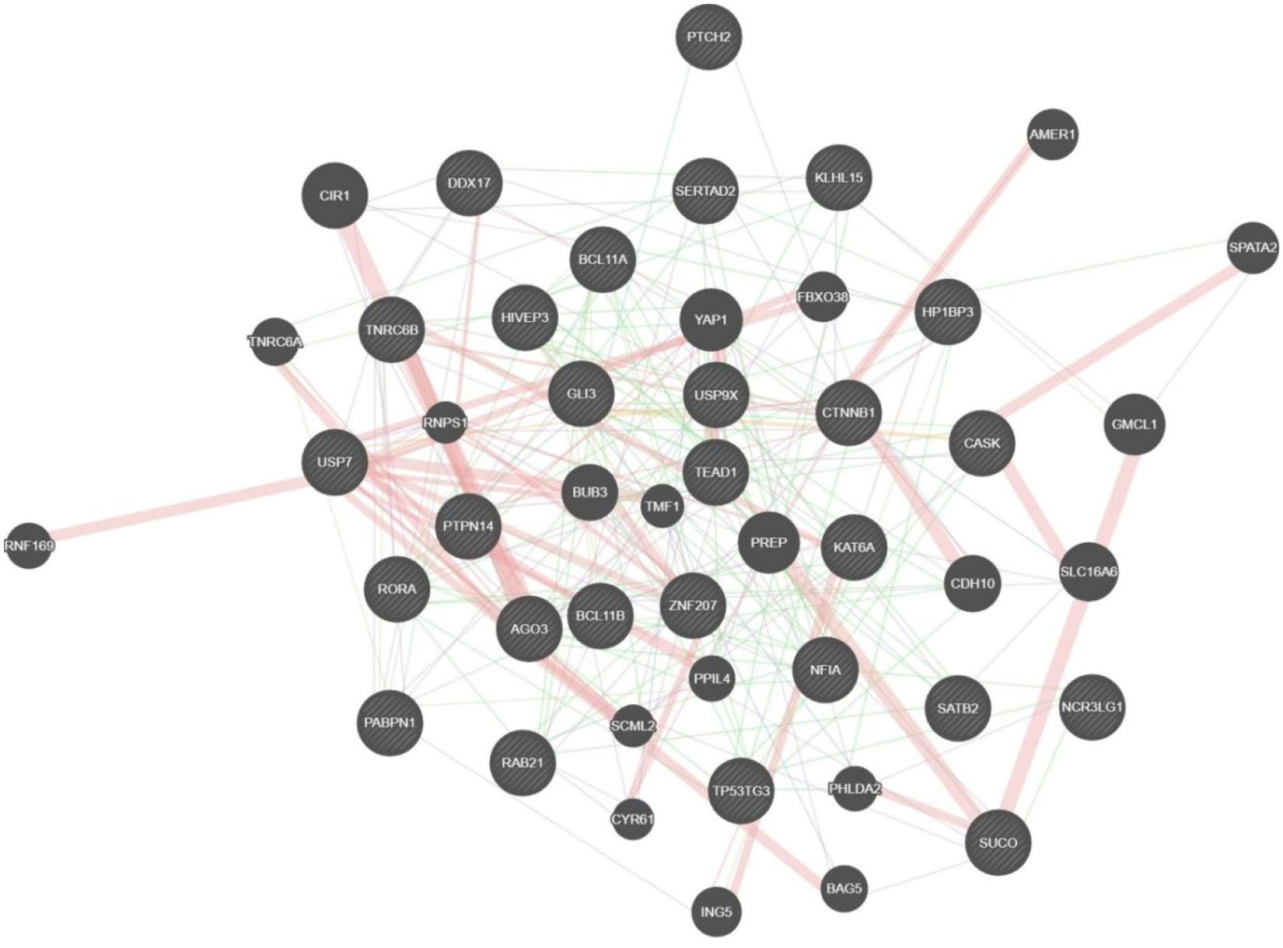
Single miRNA mode for miR-4484-Target Interaction Network. The network between targets based on Genemania tool.

To establish the function of miR-4484, we performed the functional enrichment analysis of potential gene targets, which later were analyzed by KEGG pathway, GO enrichment analysis, and protein-protein interaction analysis. The major target genes of miR-4484 involved in SSc pathogenesis and signaling pathways are summarized in (Fig. 3A). Following, using the DIANA-miR Path, we determined the biological functional interpretation of miR-4484 validated targets. Then, using KEGG and GO, we found 21 and 51 pathways, respectively, assigned for miR-4484, including the Hippo, TGF-β, ErbB signaling pathways, ECM-receptor interaction, and focal adhesion, identified as potential pathways involved in SSc pathogenesis (Fig. 3B). Moreover, the GO analysis provides the biological process, molecular function, and cellular component, which may play a role in the pathogenesis of SSc (Fig. 3C). In addition, using Genemania tool^9^, we found the interactions between the target genes, for example, YAP1, which limit TGF-β signaling along with TAZ, PTPN14, which is a negative regulator of YAP activity, AGO3, which plays a role in RNA interference, CTNNB1 – in Wnt signaling pathway and epithelial-to-mesenchymal transition or TEAD1 – in smooth muscle development (Fig. 4)^12^.

#### miR-4484 target predictions based on the gene locus and MMP-21 validation by RT-qPCR

**Figure 5:**
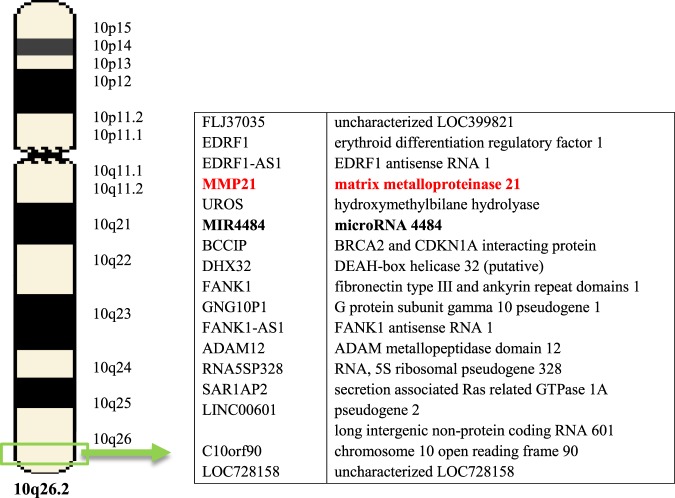
miRNA target gene predictions using gene localization on a specific chromosome.

Besides the Bioinformatics assay with available online tools, we performed the analysis of the miR-4484 target genes based on its chromosomal locus. We found that MIR4484 is an RNA gene located at chromosome 10 in locus q26.2 (Fig. 5). We paid attention to the matrix metalloproteinase 21 (MMP-21) gene since it is orientated closely to MIR4484, and the metalloproteinases are known to be involved in the fibrotic process in SSc. Since, MMP-21 has not yet been evaluated in the disease, we choose MMP-21 for further analysis. We hypothesized that miR-4484 might be involved in regulating MMP-21 expression due to close gene location, although, MMP-21 is not predicted to be a target of miR-4484 by bioinformatic programs described above. Using RT-qPCR, we validated MMP-21. The expression was significantly increased in SSc patients compared to healthy controls (*P* < 0.05; Fig. 2B).

## Discussion

The present study provided the following new findings to be discussed: (i) miRNA-4484 is differentially expressed in serum of SSc patients with 18-fold up-regulation when compared to controls; (ii) serum level of MMP-21 is significantly up-regulated in SSc serum compared to the healthy control group.

With the advance of molecular biology technologies, molecular markers of SSc have gained increasing attention. Several studies have focused on the dysregulation of miRNAs to provide their involvement in SSc pathogenesis. The first paper was published in 2011 by Kawashita *et al*. showing that miR-29a was detectable in serum of SSc patients^13^. Since then, some miRNAs have been proven as post-transcriptional regulators with dual pro- or anti-fibrotic effect in SSc. They have also been found to be associated with specific clinical characteristics, such as either skin or lung involvement, as well as disease activity and severity^13,14^.

In the present study, using miRNA microarray chip analysis comprising 30 424 total mature miRNAs probe sets, we identified that expression of 15 miRNAs is altered <−2 or >2-fold change (down- or upregulated) in our SSc patients. In particular, we found that miR-4484 was an 18-fold up-regulated in the serum of all studied SSc patients compared to healthy controls. Thus, we focused on miR-4484 in further analysis. To our knowledge, this is the first study that identifies the expression of miR-4484 in the serum of patients with SSc.

MiR-4484 is an RNA gene and has a sequence of 64-AAAAGGCGGGAGAAGCCCCA-83. It is located at chromosome 10q26.2, and it contains 83 bases. Up to now, the expression of miR-4484 has been evaluated in several types of cancer, such as breast cancer^15^, glioblastoma^16^, diffuse large B-cell lymphoma^17^, as well as oral lichen planus^18^. Interestingly, there is only one study, by Christmann *et al*. who found miR-4484 significantly down-regulated in lung tissue samples from patients with SSc-ILD (interstitial lung disease), what makes our finding more intriguing and attracting more attention in terms of a possible role of miR-4484 as a regulator of fibrotic processes in SSc^19^. Observation reported by Christmann *et al*. is in opposite to our results; however, it was rather adjunctive to the main target of the study (miR-155), and authors did not either validate target genes for miR-4484 or its expression in the blood^19^. Thus, the direct comparison with our observations might not be reliable, and the potential difference in lung and blood expression of miR-4484 might need further investigation. In our study, to predict the role of miR-4484 in SSc, we searched for its target genes associated with 3 key pathogenic events of SSc: fibrosis, vascular damage or immune dysregulation using web-based computational analysis tools. We have predicted that miR-4484 may regulate numerous potential target genes or signaling pathways, some of which with great importance in the context in the SSc development. Among others, miR-4484 may be enriched in TGF-β/Smad or Wnt/β-catenin signaling pathways, collagens expression, and regulation of MMP, as well as the tissue inhibitors of metalloproteinases (TIMP) homeostasis.

**Figure 6:**
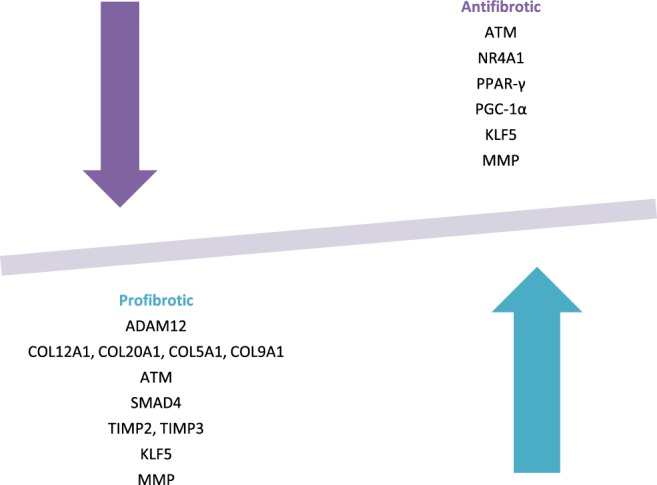
Potential target genes that may play a pro-fibrotic and/or anti-fibrotic role in SSc pathogenesis.

TGF-β and Wnt/β-catenin signaling is hyper-activated in SSc and induces Smad-dependent fibrotic responses in mesenchymal cells^20^; thus, it has a potent pro-fibrotic effect. Canonical Wnt signaling is necessary for TGF-β-mediated fibrosis, because it induces the activation and differentiation of fibroblasts and excessive collagen release^5,20^. Found in our bioinformatics analysis possible fibrosis-related targets for miR-4484 in TGF-β and Wnt/β-catenin signaling include ATM (ataxia telangiectasia mutated protein kinase), NR4A1 (nuclear receptor subfamily 4, group A, member 1), peroxisome proliferator-activated receptor gamma (PPAR-γ), coactivator 1 alpha (PGC-1α), integrin alpha 9 (ITGA9), SMAD4 and −9. Moreover, the connective tissue growth factor (CTGF) gene has been identified as a potential target for regulation by miR-4484 (Fig. 6).

ATM signaling plays a major role in the regulation of cell apoptosis, transcription, and metabolic pathways by activation of the double-strand repair pathways. The DNA damage checkpoint ATM could repress Wnt inhibitor factor 1 (WIF1) *via* the phosphorylation of the transcription factor c-Jun in SSc fibroblasts and disrupts Wnt signaling by binding directly to Wnt ligands^21,22^. The reduction of WIF1 results in Wnt pathway activation, and ultimately enhanced fibrosis in SSc^21,22^.

NR4A1 is the anti-fibrotic nuclear receptor that was shown to be elevated in SSc skin^22,23^. NR4A1 is an endogenous inhibitor of TGF-β signaling. NR4A1 recruits a repressor complex comprising SP1, SIN3A, CoREST, LSD1, and HDAC1 to TGF-β target genes, limiting pro-fibrotic TGF-β effects. Under normal wound healing conditions, TGF-β induces NR4A1 expression to stop fibroblast activation. In contrast, fibrotic conditions with persistent TGF-β activation inhibited the negative feedback mechanism by AKT- and HDAC-mediated NR4A1 repression and inactivation^22^.

PGC-1α, is a transcriptional coactivator encoded by the PPARGC1A gene, which interacts with nuclear receptor PPAR-γ. PPAR-γ has an anti-fibrotic effect mainly related to the inhibition of TGF-β/Smad signal transduction, but other pathways can be involved. PPAR-γ ligands, including PGC-1α, can directly disrupt TGF-β signal transduction and suppress TGF-β production. Its aberrant function seems to be implicated in pathological fibrosis of the skin and lungs of SSc patients^24^. On the other hand, TGF-β seems to reduce PPAR-γ expression in fibroblasts^25^. Thus, recent studies identified the role of PPAR-γ in regulating connective tissue homeostasis, with a critical role in skin and lung fibrosis of SSc^24^. Of note, another miRNAs have been found to regulate PPAR-γ activation. Lou *et al*. found that miR-130b seems to have a pro-fibrotic role in SSc by negative regulation of PPAR-γ, enhancing TGF-β signaling^22^.

SMADs proteins were characterized as TGF-β signaling intracellular effectors, which affect the phenotype of scleroderma fibroblasts^26^. SMAD4 plays a critical role in fibrosis^27^, by stimulating ECM production, including type I, III, and IV collagen, laminin, fibronectin (FN), and proteoglycans. With respect to collagens, TGF-β–induced collagen proteins were not altered in Smad4-deficient fibroblasts^28^. On the other hand, SMAD9 acts as an intracellular mediator of TGF-β signaling by represses intracellular BMP signaling as a dominant-negative Smad^29^.

Our bioinformatics analysis indicates that miR-4484 may also be involved directly in the regulation of collagen expression because COL5A1, COL9A1, COL12A1, and COL20A1 genes are possible targets and their overexpression has been found to result in increased collagen synthesis in SSc^30^. Importantly, it has been reported that the upregulation of other miRNAs may induce the Col1A1 expression (miR-21b) or other ECM molecules (miR-92a) in SSc^10^.

In terms of MMP and TIMP regulation, we found that, miR-4484 might target anti-fibrotic molecules, such as MMP8 (matrix metalloproteinase −8) and some of ADAMs (a disintegrin and metalloproteinases), including ADAM8, ADAM12, ADAM19, ADAM22, which are a family of transmembrane and secreted metalloendopeptidases of dual action in fibrosis. Of note, the potential miR-4484 target ADAM12 expression is induced by TGF-β and promotes TGF-β-dependent signaling through interaction with the type II receptor of TGF-β^27^. It has been reported that ADAM12 is overexpressed in perivascular cells in diffuse cutaneous SSc (dcSSc) and may be involved in myofibroblast trans-differentiation, implying their potential impact on the fibrotic process. Moreover, increased expression of ADAM12 has been shown in fibroblasts of dcSSc skin and lung tissue^31^. Interestingly, the serum ADAM12 levels may be associated with the initiation and progression of fibrosis, as well as the development of interstitial lung disease in SSc patients^32^.

On the other hand, in our bioinformatics analysis, we found TIMP2 and TIMP3, which are known for their pro-fibrotic properties, as direct targets for miR-4484. Their mechanism of action is inhibition of MMPs, ADAMs, and ADAMTSs, and they can directly restrict ECM proteolysis or indirectly promote ECM accumulation^33^. Thus, increased TIMPs levels results in ECM deposition (or fibrosis). Moreover, the imbalance between TIMP-2 and MMP2 shows that increased TIMP2 levels affect the inhibition of MMPs, such as collagenase, gelatinase, and stromelysin, which control the ECM metabolism, contributing to pathophysiological ECM accumulation. Also, TIMP3 has been demonstrated to regulate inflammation through inhibition of ADAM17^34^, and inhibit ECM remodeling^33^. Arpino *et al*. found that the expression of TIMP3 was higher in SSc skin fibroblasts *in vitro*, and in fibrotic skin of localized scleroderma *in vivo*^33^.

Another target for miR-4484 found in our bioinformatics analysis is the connective tissue growth factor (CTGF), which is a primary mediator of chronic fibrosis. Its expression has been found up-regulated in skin-biopsy samples and skin fibroblasts *in vitro*, as well as lung tissue from patients with SSc. In addition, CTGF may be a regulator of fibroblast proliferation and the ECM production - processes that are relevant in the course of SSc^35^.

We also found that miR-4484 may potentially regulate the KLF5 (Kruppel-like factor 5) gene, which attracted much attention in the past few years in terms of epigenetics of SSc. This gene encodes a member of the Kruppel-like factor subfamily of zinc finger proteins. It is broadly expressed in skin, and it may participate in both promoting or suppressing cell proliferation^36^. Moreover, the study shows that KLF5 is similar to FLI1, and it was epigenetically suppressed by hypermethylation at its promoter region and H3/H4 hypoacetylation in SSc fibroblasts^22,37^. Moreover, the two transcription factors deficiency in animals led to enhanced fibrosis, vascular pathology in skin and lungs, and dysfunction of the immune system, implicating the role of KLF5 in SSc pathogenesis^22,37^. Additionally, importantly for SSc-related vasculopathy, KLF5 promotes angiogenesis through directly regulating VEGFA transcription^38^. Of note, KLF5 has been found to contain the target site of several miRNAs, including miR-375, miR-124, miR-506, miR-5195-3p, miR-217, and its expression may be post-translationally modified (mainly suppressed or down-regulated) by those miRNA [https://www.ncbi.nlm.nih.gov/gene/688].

The second novel finding in our study is the demonstration of significant upregulation of MMP-21 in the serum of SSc patients. MMP-21 is a member of the metalloproteinase superfamily that is known to hydrolyze ECM components^39^. MMP-21 is cytoplasmic protein and not present on the cell surface^40^. MMP-21 is expressed in numerous normal, malignant, and fetal tissues^41^. In addition, MMP-21 expression is mostly epithelial and, importantly, MMP-21 is expressed by suprabasal differentiating keratinocytes in developing skin; however, it is probably not present in healthy adult skin^39,42^. In chronic wounds, MMP-21 protein was detected on the surface of the wounds, in a subpopulation of macrophages and activated fibroblasts^40,43^. Up to now, there is no convincing data according to the role of MMP-21 in fibrotic processes, and it was not reported in SSc or other CTDs^43^. Skoog *et al*. found that MMP-21 expression is downregulated by TGF-β in the skin and cultured fibroblasts, and they indirectly concluded that lack of MMP-21 might contribute to skin fibrosis, and connective tissue remodeling^40^. However, on the contrary, Ahokas *et al*. have found MMP-21 to be up-regulated by TGF-β in mice keratinocytes^42^. Moreover, MMP-21 protein was found *in vivo* in fibroblasts in dermatofibromas; thus, it may affect the growth pattern of these lesions. Additionally, the presence of the T-cell factor-4 (Tcf-4) motif, suggests that the MMP-21 promoter may be a target of the Wnt signaling pathway, which interacts with TGF-β1 pathways^44^. Importantly, it may also act as a negative regulator of NOTCH-signaling pathway^45^ and cleaves α1-antitrypsin^39^. These findings suggest the potential regulatory role of MMP-21 in fibrotic events, including SSc, but it requires further validation. Moreover, based on the role of MMP-21 as a collagenase-4^46^, it is intriguing to hypothesize that it may play a role in the degradation of type IV collagen of the vessel basement membrane, thus potentially leading to SSc related microvascular injury.

Although MMP-21 is not predicted to be a target of miR-4484 by bioinformatics programs described above, it has attracted our attention since miR-4484, and MMP-21 genes are located in the immediate vicinity on chromosome 10, and MMP-21 has not been evaluated in SSc yet. miR-4484 is an intragenic miRNA that resides in the first intron of the uroporphyrinogen III synthetase (UROS) gene and MMP-21 resides downstream to UROS (at the distance of ~13 kb)^16^. Based on this data, it is tempting to speculate whether there exists any inter-relationship between the expression of miR-4484 and MMP-21^16^. In our study, similarly to miR-4484, MMP-21 has been found over-expressed in SSc serum, thus a concomitant expression of both genes induced by other, yet unknown, factors seems reasonable. However, in the study by Nawaz *et al*. there was a significant positive correlation in expression of miR-4484 and UROS, but not of MMP-21 in glioblastoma^16^. Consequently, it might be rather assumed that increased expression of miR-4484 may potentially up-regulate MMP-21 on the post-transcriptional level (enhancement of translation), thus, promoting its production. Since MMP-21 is not predicted to be a target of miR-4484 by bioinformatics programs, miR-4484 might regulate MMP-21 expression also indirectly through other targets. Since MMP-21 may contribute to the excess collagen deposition in SSc by decreasing collagen degradation, it might be assumed that an increased level of miR-4484 may play a role in the pathogenesis of SSc also through the up-regulation of MMP-21. To sum up whether and how miR-4484 may regulate expression of MMP-21 or it is only co-incidental remains to elucidate in further studies. Summary of possible implications in SSc pathogenesis of targets for miR-4484 is shown in Table 2.

**Table 2:** Summary of possible implications in SSc pathogenesis of targets for miR-4484.

Gene		Implications	Ref.
ADAM8, ADAM12, ADAM19, ADAM22	ADAM metallopeptidase domain 8, 12, 19, 22	Possibly contributing to fibrosis through the TGF-β pathway	^27^
MMP-8	Matrix metallopeptidase 8	Degradation of ECM components Role in cell proliferation, migration, apoptosis and angiogenesis	
COL5A1, COL9A2, COL12A1, COL20A1	Collagen: type V, α1 type IX, α2 type XII, α1 type XX, α1	Overexpression of collagen	^30^
DDX3×	DEAD (Asp-Glu-Ala-Asp) box helicase 3	X-chromosome genes	
ITGA9	Integrin, alpha 9	Possibly involved in myofibroblast transformation and TGF-β regulation overexpressed in both dcSSc and lcSSc fibroblasts	^22^
KLF5	Kruppel-like factor 5 (intestinal)	KLF5 expression; downregulation of KLF5 and FLI1 work synergistically to exacerbate fibrosis	^22^
MYO1	Myosin IE	Involved in actin assembly; role in SSc unclear	^22^
NR4A1	Nuclear receptor subfamily 4, group A, member 1	Acetylation occurs at NR4A1 promoter in the presence of TGF-β	^22^
YAP1	Yes-associated protein 1	Limit TGF-β signaling along with TAZ, further suggesting that inhibiting YAP–TAZ nuclear translocation might offer a new therapeutic approach for fibrosis	^61^
YBX1	Y box binding protein 1	Involved in Notch signaling	
SMAD4, SMAD9	SMAD family	TGF-β modulators Involved in cell signaling	^29^
IL-1β, IL-6 IFN-γ TNF TGF-β, TGF-βR	Interleukins Interferon Tumor necrosis factor Transforming growth factor	Cytokines and growth factors	^20^
CCL4, CCL5, CXCL10		Chemokines	
CD4, CD8		T-lymphocytes	
TIMP2, TIMP3	TIMP metallopeptidase inhibitor	MMPs inhibitors	^33^
CTGF	Connective tissue growth factor	Modular secreted protein Angiogenesis and wound healing	^35^

Of note, our study has limitations. A group of patients was small, and only female patients were enrolled; thus, results should ideally be validated in a larger group involving both sexes. Due to the limited number of SSc patients, we could not examine the correlations of both miR-4484 and MMP-21 with specific clinical and laboratory features of SSc. Moreover, the measurement of miRNAs as a biomarker is associated with some challenges, such as pre-analytic variation and data analysis. Also, the choice of the biological fluid may have an impact on miRNAs profiling since the contamination of intracellular RNA from platelets or erythrocytes could introduce bias. For example, there is a difference of miR-142-3p levels assayed in SSc plasma and serum^21,27,46^. In addition, changes in non-coding RNAs in tissue may not reflect similar changes in circulation, adding to the difficulties in data interpretation^22^.

## Conclusion

In conclusion, we have identified that miR-4484 is significantly up-regulated in the serum of SSc patients, thus arising a candidate for diagnosing and elucidating the pathogenesis of the disease worth further functional analysis. Considering a number of potential miR-4484 target genes of molecules implicated in fibrotic processes, a pathogenic role of miR-4484 in SSc is highly possible, however yet unclear. Further investigation, whether miR-4484 and/or MMP-21 can modulate fibrotic response may be substantial. Moreover, identification of the regulatory mechanisms of matrix metalloproteinases, including MMP-21 and collagen expression by miR-4484 may also lead to the new therapeutic tool using miRNAs by the transfection into fibrotic lesions.

## Methods

### Patients

The study enrolled 10 SSc female patients (aged 43–85, mean ± SD: 63.10 ± 12.53 years), referred to the Department of Dermatology, Medical University of Lublin, Poland over a 2 years. All patients fulfilled the classification criteria of the 2013 ACR (American College of Rheumatology)/EULAR for SSc^47^. Patients with overlap syndromes, cardiovascular diseases, diabetes mellitus, hyperlipidemia, thrombosis, pregnancy, neoplastic diseases, and those with habitual cigarette smoking and alcohol drinking were excluded from this study. Moreover, all patients were on a stable treatment regimen for at least 6 months. The control group consisted of 6 healthy age- and sex-matched subjects with no medical history (Table 3). The study protocol was following the Helsinki Declaration of 1975 (revised in 2000) and was approved by the Bioethics Committee of the Medical University of Lublin. All individuals gave written informed consent to participate in the study.

**Table 3:** Clinical characteristics of studied patients with systemic sclerosis (SSc) and healthy controls.

Parameters	SSc patients (n = 10)	Healthy controls (n = 6)
Female/male ratio	10/0	6/0
Age [years], mean ± SD (range)	63.10 ± 12.53 (43–85)	57.25 ± 5.62 (50–60) *P* < 0.02
Disease subset:		
Limited (lcSSc), n(%)	8 (80%)	
Diffuse (dcSSc), n(%)	2 (20%)	
mRSS, mean ± SD (range)	9.70 ± 4.76 (2–20)	
Digital ulcers (DUs):		
Active digital ulcers, n(%)	3 (30%)	
No active digital ulcers, n(%)	7 (70%)	
Interstitial lung disease (ILD), n(%)	10 (100%)	
Anti-nuclear antibodies:		
ANA positive, n(%)	10 (100%)	
anti-Scl-70 positive, n(%)	4 (40%)	
ACA positive, n(%)	5 (50%)	
anti-polymerase III, n(%)	1 (10%)	
CRP (mg/L) (range)	5.64 ± 6.61 (1–20)	
OB (mm/h) (range)	24.10 ± 16.79 (2–50)	
HRCT	10 (100%)	
DCO(SB), mean ± SD (range)	72.50 ± 31.88 (28–114)	
TLC, mean ± SD (range)	90.50 ± 22.58 (0–127)	

SSc, systemic sclerosis; lcSSc, limited cutaneous SSc; dcSSc, diffuse cutaneous SSc; mRSS, modified Rodnan skin score; DUs, digital ulcers; ILD, interstitial lung disease; ANA, anti-nuclear antibodies; ACA, anti-centromere antibodies; Scl-70, anti-topoisomerase I antibodies; HRCT, high resolution computed tomography; DCO(SB), diffusing capacity for carbon monoxide, single-breath-measurements; TLC, total lung capacity.

### Clinical and laboratory assessment

According to criteria proposed by LeRoy, patients with SSc were classified into two groups, limited cutaneous SSc (lcSSc) or diffuse cutaneous SSc (dcSSc)^48^. C-reactive protein concentration and erythrocyte sedimentation rate (ESR) were measured and used as a marker of inflammation. Skin involvement was assessed using the modified Rodnan skin score (mRSS) as described elsewhere^49^. Lung function was assessed using pulmonary function tests, including measurements of the forced vital capacity (FVC) and diffusing capacity of the lungs for carbon monoxide (DLCO)^50^. Using a color Doppler echocardiography, the pulmonary artery pressure was measured^50^. Based on the presence of reticular and/or ground-glass opacification on high-resolution computed tomography (HRCT) scans, the presence of interstitial lung disease was defined^50^. Scleroderma renal crisis was defined as a new onset of renal failure with or without a marked increase in systemic blood pressure, which could not be explained by other reasons^49^. Moreover, digital ulcers (DUs) were defined as painful areas of tissue loss located on the volar surface of the fingertips or around the nail distal to the proximal interphalangeal digital crease^49^. Antinuclear antibodies (ANA) and anti-centromere antibodies (ACA) were determined by using indirect immunofluorescence on HEp-2 cells and anti-topoisomerase I (Scl-70), anti-polymerase III antibodies by immunoblotting analysis^50^.

### miRNA isolation

Whole blood (5–10 mL) was collected in BD Monovette plastic tubes (Sarstedt, Germany). The samples were stored on ice and processed within 1 hour of the draw. Serum was isolated by centrifugation at 3 000 x g for 10 min at 4 °C and stored at −80 °C, in the absence of freeze-thaw cycles, until analysis. Before RNA extraction, we checked the serum samples to test for hemolysis by measuring the absorbance of free hemoglobin at 414 nm, and samples with OD_414_ greater than 0.2 were excluded^51^, due to the potential of cellular miRNAs to confound the results. RNA was extracted from 100 µL serum using the miRNeasy Serum/Plasma Kit (Qiagen, Germany) following the manufacturer’s protocol. Briefly, after Qiazol solution (1 mL) was added and mixed by vortexing, samples were incubated at room temperature for 5 min, the addition of chloroform was useful to achieve aqueous and organic phase separation. In the next step, the aqueous phase was applied to an RNeasy spin column. The purified RNA was eluted in 14 µL RNase-free water, split into single-use aliquots and stored at −80 °C.

### Analysis of miRNA quality and integrity

miRNAs concentration, A260/230, and A260/280 ratios were evaluated by NanoDrop UV/Vis spectrophotometer (2000, Thermo Fisher Scientific, Waltham, MA, USA). Later, the quality and the related size of total and small RNA was measured by capillary electrophoresis with 2100 Agilent Bioanalyzer (Agilent Technologies, Santa Clara, CA, USA) using two different chips: Agilent RNA 6000 Nano Kit for total RNA and Agilent Small RNA kit for low molecular weight RNA. Electropherograms were visualized with the Agilent 2100 Expert software.

### Analysis of miRNA quantity using synthetic miRNA standards

Because the assessing of the ratio of miRNA to total RNA is challenging, we used the fixed volume of serum, rather than a fixed miRNA amount for RT-qPCR as recommended in several studies^52,53^. In addition, to check the variations in RNA extraction, we routinely spike-in non-human (*C*. *elegans*) synthetic miRNAs to the samples at the lysis buffer step before the RNA extraction using the miScript reverse transcription kit (Qiagen, Germany) and miScript SYBR Green PCR kit (Qiagen, Germany) following the manufacturer’s protocol. Briefly, in 1 mL PCR tubes, 5 µL RNA was added to 15 µL master mix, consisting of 4 µL 5x HiSpec Buffer, 2 µL 10x Nucleics Mix, 2 µL RT Mix and 7 µL nuclease-free water. All tubes were incubated at 37 °C for 1 h. cDNA was diluted 1:11 in nuclease-free water and stored at −80 °C. Reactions were performed in the CFX96 real-time PCR detection system (Bio-Rad, Hercules, CA, USA) at 60 °C for 60 min, followed by 95 °C for 5 min and holding at 4 °C. Reactions containing nuclease-free water in place of the RNA template served as negative controls. The generated standard curve was used to estimate the absolute copy number of the target miRNA in biological samples.

### Microarray procedures and data analysis

All serum RNA isolations assessed by microarray following the manufacturer’s protocols. Briefly, RNA samples were obtained from starting material (200 µL of serum), then a total of 16 samples from patients and healthy controls were labeled using the FlashTag™ Biotin HSR RNA Labeling Kit (Affymetrix, Santa Clara, CA, USA) and hybridized overnight to the Affymetrix GeneChip miRNA array (P/N 901326). The arrays were washed and stained using standard Affymetrix protocols and scanned using the Affymetrix GCS 3000 7 G Scanner. Feature intensities were extracted using the miRNA_4.0 library files. For microarray data analysis, Affymetrix Transcriptome Analysis Console (TAC) Software v4.0 was used. The quality control and data normalization (probe set normalization) were assessed according to Affymetrix indications for the FlashTag™ Biotin HSR RNA Labeling Kit. Differentially expressed miRNAs were identified based on RVM *t*-test analysis. Differentially expressed miRNAs with at least 2-fold change in either direction with *P* < 0.05 were considered being up- or downregulated.

The miRNA expression data has been deposited in NCBIs Gene Expression Omnibus (GEO, http://www.ncbi.nlm.nih.gov/geo/) and are accessible through the GEO series accession number GSE137472.

### Quantitative RT-PCR (RT-qPCR) of selected miRNAs and MMP-21

miRNAs expression levels were measured in all 16 samples by RT-qPCR using the TaqMan MicroRNA Reverse Transcription Kit (Applied Biosystems, Foster City, CA, USA). In the first step, total RNA (25 ng) was used for reverse transcription. From 15 µL of RT mixture, 1.33 µL was used for RT-qPCR, which was performed using TaqMan Universal PCR Master Mix (Applied Biosystems) and TaqMan probes (Applied Biosystems) in 96-well plates using the CFX96 real-time PCR detection system (Bio-Rad, Hercules, CA, USA) following the manufacturer’s protocol. Specific miRNAs primers were used for miR-4484, miR-584, and miR-4529-3p (Life Technologies, Carlsbad, CA, USA), as well as the RNU48 as the control for miRNA normalization (Life Technologies).

Moreover, RT-qPCR in all 16 samples was performed to verify the expression of MMP-21 by TaqMan RNA-to-Ct 1-Step Kit (Applied Biosystems). Specific mRNA primers were used for metalloproteinase-21 (MMP-21). Actin (ACTN) served as the control for mRNA normalization (Life Technologies). Gene expression was analyzed using the difference in cycle threshold (ΔCt) method using the manufacturer’s software (Bio-Rad). The Ct values were normalized to RNU6B for miRNAs and to ACTN for mRNA. All PCR reactions were run in 3 replicates. The relative miRNAs/mRNA expression levels were calculated using the 2^−ΔΔCt^ method (following equation: relative gene expression = 2−(ΔCt sample − ΔCt control).

### Pathway and network analysis

#### Target gene prediction

Relevant target genes were identified by using databases Target Scan v7.1 (http://www.targetscan.org)^54^, which predicts miRNAs biological targets based on the conserved 8-, 7-, and 6-mer sites matching the seed region of each miRNA^55^, miRDB v4.0 (http://mirdb.org/miRDB/)^56^, miRTarBase (http://mirtarbase.mbc.nctu.edu.tw)^49^, and TargetMiner (http://www.isical.ac.in/~bioinfo_miu/targetminer20.htm). Another used tool was miRSearch v3.0 (https://www.exiqon.com/miRSearch), which also finds relevant miRNAs, which uses an advanced algorithm to cross-reference all annotations so that a comprehensive list of microRNA-mRNA interactions can be displayed (https://www.exiqon.com/miRSearch).

Moreover, we performed pathway analysis by DNA Intelligent Analysis (DIANA)-miRPath v5.0 software, based on data from Ensembl v69 and miRBase v18 (http://diana.imis.athena-innovation.gr)^57^, which uses miRNA targets based on DIANA-microT-CDS, and predicts miRNA-gene interaction including the binding region, position, and type, and/or miRNA-gene interaction experimentally validated from TarBase v7.0^57^.

#### Enrichment analysis

Bioinformatics analysis for Gene Ontology (GO) term enrichment (http://www.geneontology.org)^58^ was performed to assess the molecular function, biological processes, molecular functions and cellular components, disease association, and gene tissue expression associated with the target genes of hsa-miR-4484 predicted by Target Scan. Moreover, we used the Database for Annotation, Visualization and Integrated Discovery (DAVID v6.7; https://david.ncifcrf.gov), and the Kyoto Encyclopedia of Genes and Genomes database (KEGG v58; http://www.genome.jp/kegg)^59^.

The DAVID 6.7 can map numerous interesting genes associated with GO terms, showing the most over-represented (enriched). The Protein IDs of the validated targets of miR-4484 were converted into gene lists and uploaded into the DAVID database. Functional categories were enriched within genes that were differentially expressed between the SSc group and a control group. In addition, the KEGG was used to identify the miRNA targets associated with signaling pathways in SSc. *P* < 0.05 adjusted by the Benjamini-Hochberg procedure indicates significance.

### Statistical analysis

Data analysis was performed using GraphPad Prism v6.0a software (GraphPad Software, San Diego, CA, USA). Data are expressed as mean ± SD. Significance of RT-qPCR experiments was determined by one-way analysis of variance (GraphPad Prism Software, San Diego, CA, USA). Therefore, two-group comparisons were performed with Student’s *t*-test or the Mann-Whitney test. Comparisons of multiple groups were analyzed using two-way analysis of variance (ANOVA) followed by Tukey’s test. Values of *P* < 0.05 were considered statistically significant. Microarray data were analyzed by subtracting the background, and normalizing the signals using the Median Normalization Method, where normalized Data is equal to division of the subtraction of foreground and background, and median^60^. Thus, the median represents 50% of miRNAs intensity, an intensity that is larger than 50 in all samples after background correction^60^. After normalization, the statistical significance of differently expressed miRNAs was analyzed by Student’s *t-*test. Hierarchical clustering and correction analysis were performed on miRNAs data.

## Supplementary information

Dataset 1

Dataset 2

## Data Availability

The datasets generated and analyzed during the current study are available in the GEO repository, series accession number GSE137472.
