# Supplementary material for: A novel miRNA-4484 is up-regulated on microarray and associated with increased MMP-21 expression in serum of systemic sclerosis patients
Source: Sci Rep. 2019 Oct 3;9:14264. doi: 10.1038/s41598-019-50695-y (PMC6776520; doi:10.1038/s41598-019-50695-y)
Supplement: Supplementary file 1 — Dataset 1 [file 41598_2019_50695_MOESM1_ESM.pdf]

A novel miRNA-4484 is up-regulated on microarray and associated with increased MMP-21 expression in serum of systemic sclerosis patients

Marta Rusek[1,2,§,*], Małgorzata Michalska-Jakubus[1,§], Małgorzata Kowal[1], Jerzy Bełtowski[2], Dorota Krasowska[1]

[1]Department of Dermatology, Venereology and Pediatric Dermatology, Laboratory for Immunology of Skin Diseases, Medical University of Lublin, Lublin, Poland

[2]Department of Pathophysiology, Medical University of Lublin, Lublin, Poland

Supplementary Table 1. Predicted targets for hsa-miR-4484 in Target Scan, miRSearch, miRDB, miRTarBase, DIANA-microT databases, which may play a role in SSc pathogenesis

Gene target	Representative transcript	Gene name	Software
**AATF**	**ENST00000225402.5**	apoptosis antagonizing transcription factor	Target Scan, miRSearch
**ADAM19**	**ENST00000257527.4**	ADAM metallopeptidase domain 19	Target Scan
**ADAM22**	**ENST00000398204.4**	ADAM metallopeptidase domain 22	Target Scan
**ADAM8**	**ENST00000445355.3**	ADAM metallopeptidase domain 8	Target Scan, miRSearch
**ADAMTS20**	**ENST00000389420.3**	ADAM metallopeptidase with thrombospondin type 1 motif, 20	Target Scan
**ADAMTS4**	**ENST00000367996.5**	ADAM metallopeptidase with thrombospondin type 1 motif, 4	Target Scan
**ADAMTS5**	**ENST00000284987.5**	ADAM metallopeptidase with thrombospondin type 1 motif, 5	Target Scan
**ADCY3**	**ENST00000260600.5**	adenylate cyclase 3	Target Scan, miRDB
**AGO3**	**ENST00000373191.4**	argonaute RISC catalytic component 3	miRDB, DIANA-microT
**AK4**	**ENST00000545314.1**	adenylate kinase 4	Target Scan, miRTarBase
**ANKRD23**	**ENST00000318357.4**	ankyrin repeat domain 23	Target Scan, miRTarBase
**ANLN**	**ENST00000265748.2**	anillin, actin binding protein	Target Scan, miRSearch
**ANO1**	**ENST00000355303.5**	anoctamin 1, calcium activated chloride channel	Target Scan, miRDB
**APP**	**ENST00000346798.3**	amyloid beta (A4) precursor protein	Target Scan, miRDB, miRTarBase
**BCL2L2-PABPN1**	**ENST00000553781.1**	BCL2L2-PABPN1 readthrough	Target Scan, miRDB, miRTarBase
**CADM1**	**ENST00000452722.3**	cell adhesion molecule 1	Target Scan, miRTarBase
**CDC42SE1**	**ENST00000439374.2**	CDC42 small effector 1	Target Scan, miRTarBase
**CDON**	**ENST00000392693.3**	cell adhesion associated, oncogene regulated	Target Scan, miRTarBase
**CERS2**	**ENST00000368954.5**	ceramide synthase 2	Target Scan, miRTarBase
**COL12A1**	**ENST00000322507.8**	collagen, type XII, alpha 1	Target Scan
**COL20A1**	**ENST00000422202.1**	collagen, type XX, alpha 1	Target Scan
**COL5A1**	**ENST00000371817.3**	collagen, type V, alpha 1	Target Scan
**COL9A2**	**ENST00000372748.3**	collagen, type IX, alpha 2	Target Scan
**CTC1**	**ENST00000315684.8**	CTS telomere maintenance complex component 1	Target Scan, miRTarBase
**CTGF**	**ENST00000367976.3**	connective tissue growth factor	Target Scan
**CTXN1**	**ENST00000318978.4**	cortexin 1	Target Scan, miRTarBase
**DDX17**	**ENST00000396821.3**	DEAD (Asp-Glu-Ala-Asp) box helicase 17	miRDB, DIANA-microT
**DDX3X**	**ENST00000399959.2**	DEAD (Asp-Glu-Ala-Asp) box helicase 3, X-linked	Target Scan
**EFNA1**	**ENST00000368407.3**	ephrin-A1	Target Scan, miRTarBase
**EGFR**	**ENST00000275493.2**	epidermal growth factor receptor	Target Scan
**EIF4E**	**ENST00000450253.2**	eukaryotic translation initiation factor 4E	Target Scan, miRDB
**FBXO34**	**ENST00000313833.4**	F-box protein 34	Target Scan, miRDB
**FGF1**	**ENST00000360966.5**	fibroblast growth factor 1 (acidic)	Target Scan

**FZD4**	**ENST00000531380.1**	frizzled family receptor 4	Target Scan
**GABARAP**	**ENST00000302386.5**	GABA(A) receptor-associated protein	Target Scan, miRTarBase, DIANA-microT
**GATAD1**	**ENST00000287957.3**	GATA zinc finger domain containing 1	Target Scan, miRTarBase
**GLIS3**	**ENST00000324333.10**	GLIS family zinc finger 3	Target Scan, DIANA-microT
**GXYLT1**	**ENST00000398675.3**	glucoside xylosyltransferase 1	Target Scan, miRTarBase
**HAVCR1**	**ENST00000522693.1**	hepatitis A virus cellular receptor 1	Target Scan, miRTarBase, DIANA-microT
**HOXC5**	**ENST00000312492.2**	homeobox C5	Target Scan, miRDB
**HSPA2**	**ENST00000394709.1**	heat shock 70kDa protein 2	Target Scan
**IGLON5**	**ENST00000270642.8**	IgLON family member 5	miRDB, DIANA-microT
**IL17D**	**ENST00000304920.3**	interleukin 17D	Target Scan
**INO80D**	**ENST00000403263.1**	INO80 complex subunit D	Target Scan miRTarBase
**ITGA9**	**ENST00000264741.5**	integrin, alpha 9	Target Scan
**KIF2C**	**ENST00000372224.4**	kinesin family member 2C	Target Scan, miRTarBase, DIANA-microT
**KLF5**	**ENST00000377687.4**	Kruppel-like factor 5 (intestinal)	Target Scan
**KLHL15**	**ENST00000328046.8**	kelch-like family member 15	Target Scan, DIANA-microT
**KLHL29**	**ENST00000486442.1**	kelch-like family member 29	Target Scan, miRDB
**KPNB1**	**ENST00000290158.4**	karyopherin (importin) beta 1	Target Scan, miRTarBase
**KRT80**	**ENST00000313234.5**	keratin 80	Target Scan, miRSearch
**LAMB3**	**ENST00000356082.4**	laminin, beta 3	Target Scan, miRSearch
**LAMC1**	**ENST00000258341.4**	laminin, gamma 1 (formerly LAMB2)	Target Scan, miRTarBase, DIANA-microT
**LIMD1**	**ENST00000273317.4**	LIM domains containing 1	Target Scan, miRTarBase, DIANA-microT
**LITAF**	**ENST00000413364.2**	lipopolysaccharide-induced TNF factor	Target Scan, miRTarBase
**LRCH3**		leucine-rich repeats and calponin homology (CH) domain containing 3	miRSearch
**LRRC45**	**ENST00000306688.3**	leucine rich repeat containing 45	Target Scan, miRTarBase
**MAPKAPK5**	**ENST00000550735.2**	mitogen-activated protein kinase-activated protein kinase 5	Target Scan, miRTarBase
**MED1**	**ENST00000300651.6**	mediator complex subunit 1	Target Scan, miRSearch
**MMP8**	**ENST00000236826.3**	matrix metallopeptidase 8 (neutrophil collagenase)	Target Scan
**MSRB3**	**ENST00000308259.5**	methionine sulfoxide reductase B3	Target Scan, miRDB
**MTHFD2**	**ENST00000394053.2**	methylenetetrahydrofolate dehydrogenase (NADP+ dependent) 2, methenyltetrahydrofolate cyclohydrolase	Target Scan, miRDB
**MYCN**	**ENST00000281043.3**	v-myc avian myelocytomatosis viral oncogene neuroblastoma derived homolog	Target Scan, miRDB

**MYO1E**	**ENST00000288235.4**	myosin IE	Target Scan
**NDUFV3**	**ENST00000340344.4**	NADH dehydrogenase (ubiquinone) flavoprotein 3, 10kDa	Target Scan, miRTarBase, DIANA-microT
**NFIA**	**ENST00000403491.3**	nuclear factor I/A	Target Scan, DIANA-microT
**NPM1**	**ENST00000393820.2**	nucleophosmin (nucleolar phosphoprotein B23, numatrin)	Target Scan, miRTarBase, DIANA-microT
**NR4A1**	**ENST00000394824.2**	nuclear receptor subfamily 4, group A, member 1	Target Scan
**PABPN1**	**ENST00000397276.2**	poly(A) binding protein, nuclear 1	Target Scan, miRDB miRTarBase, DIANA-microT
**PEBP1**	**ENST00000261313.2**	phosphatidylethanolamine binding protein 1	Target Scan, miRTarBase
**PIK3IP1**	**ENST00000441972.1**	phosphoinositide-3-kinase interacting protein 1	Target Scan
**PITPNM2**	**ENST00000280562.5**	phosphatidylinositol transfer protein, membrane-associated 2	Target Scan, miRDB
**PLEKHA8**	**ENST00000449726.1**	pleckstrin homology domain containing, family A (phosphoinositide binding specific) member 8	Target Scan, miRTarBase
**PPARGC1A**	**ENST00000264867.2**	peroxisome proliferator-activated receptor gamma, coactivator 1 alpha	Target Scan
**PSD3**	**ENST00000327040.8**	pleckstrin and Sec7 domain containing 3	Target Scan, miRSearch
**PTCH2**	**ENST00000447098.2**	patched 2	Target Scan, miRDB, miRTarBase
**PTGES**	**ENST00000340607.4**	prostaglandin E synthase	Target Scan
**PTPN14**	**ENST00000366956.5**	protein tyrosine phosphatase, non-receptor type 14	Target Scan, miRTarBase, DIANA-microT
**PTPRF**	**ENST00000372414.3**	protein tyrosine phosphatase, receptor type, F	Target Scan, miRTarBase
**R3HDM1**	**ENST00000264160.4**	R3H domain containing 1	miRSearch, miRDB
**RBM28**	**ENST00000223073.2**	RNA binding motif protein 28	Target Scan, miRTarBase
**RORA**	**ENST00000335670.6**	RAR-related orphan receptor A	Target Scan, DIANA-microT
**RPA1**	**ENST00000254719.5**	replication protein A1, 70kDa	miRSearch, miRDB
**RPL14**	**ENST00000416518.1**	ribosomal protein L14	Target Scan, miRTarBase
**SACM1L**	**ENST00000389061.5**	SAC1 suppressor of actin mutations 1-like (yeast)	miRSearch, miRDB
**SATB2**	**ENST00000417098.1**	SATB homeobox 2	Target Scan, DIANA-microT
**SERBP1**	**ENST00000370994.4**	SERPINE1 mRNA binding protein 1	Target Scan
**SERINC5**	**ENST00000512721.1**	serine incorporator 5	Target Scan, miRTarBase, DIANA-microT

**SERTAD2**	**ENST00000313349.3**	SERTA domain containing 2	Target Scan, DIANA-microT
**SHC2**	**ENST00000264554.6**	SHC (Src homology 2 domain containing) transforming protein 2	Target Scan, miRSearch
**SHC3**	**ENST00000375835.4**	SHC (Src homology 2 domain containing) transforming protein 3	Target Scan, miRDB
**SLC30A7**	**ENST00000370112.4**	solute carrier family 30 (zinc transporter), member 7	Target Scan, miRTarBase, DIANA-microT
**SMAD4**	**ENST00000398417.2**	SMAD family member 4	Target Scan
**SMAD9**	**ENST00000399275.2**	SMAD family member 9	Target Scan
**SMG7**	**ENST00000367537.3**	SMG7 nonsense mediated mRNA decay factor	Target Scan, miRSearch
**SULT4A1**	**ENST00000330884.4**	sulfotransferase family 4A, member 1	miRSearch, miRDB
**THOC5**	**ENST00000490103.1**	THO complex 5	Target Scan, miRSearch
**THSD4**	**ENST00000355327.3**	thrombospondin, type I, domain containing 4	Target Scan, miRTarBase, DIANA-microT
**TIMP2**	**ENST00000585421.1**	TIMP metallopeptidase inhibitor 2	Target Scan
**TIMP3**	**ENST00000266085.6**	TIMP metallopeptidase inhibitor 3	Target Scan
**TRAK2**	**ENST00000332624.3**	trafficking protein, kinesin binding 2	miRSearch, miRDB
**UCK2**	**ENST00000367879.4**	uridine-cytidine kinase 2	Target Scan, miRTarBase, DIANA-microT
**YAP1**	**ENST00000282441.5**	Yes-associated protein 1	miRTarBase, DIANA-microT
**YBX1**	**ENST00000321358.7**	Y box binding protein 1	miRSearch, miRDB
